# Emerging Roles in the Biogenesis of Cytochrome *c* Oxidase for Members of the Mitochondrial Carrier Family

**DOI:** 10.3389/fcell.2019.00003

**Published:** 2019-01-31

**Authors:** Oluwaseun B. Ogunbona, Steven M. Claypool

**Affiliations:** ^1^Department of Physiology, School of Medicine, Johns Hopkins University, Baltimore, MD, United States; ^2^Department of Pathology & Laboratory Medicine, School of Medicine, Emory University, Atlanta, GA, United States

**Keywords:** ADP/ATP carrier, cytochrome *c* oxidase, mitochondrial carrier family, mitochondrial translation, respiratory supercomplexes, solute carrier family

## Abstract

The mitochondrial carrier family (MCF) is a group of transport proteins that are mostly localized to the inner mitochondrial membrane where they facilitate the movement of various solutes across the membrane. Although these carriers represent potential targets for therapeutic application and are repeatedly associated with human disease, research on the MCF has not progressed commensurate to their physiologic and pathophysiologic importance. Many of the 53 MCF members in humans are orphans and lack known transport substrates. Even for the relatively well-studied members of this family, such as the ADP/ATP carrier and the uncoupling protein, there exist fundamental gaps in our understanding of their biological roles including a clear rationale for the existence of multiple isoforms. Here, we briefly review this important family of mitochondrial carriers, provide a few salient examples of their diverse metabolic roles and disease associations, and then focus on an emerging link between several distinct MCF members, including the ADP/ATP carrier, and cytochrome *c* oxidase biogenesis. As the ADP/ATP carrier is regarded as the paradigm of the entire MCF, its newly established role in regulating translation of the mitochondrial genome highlights that we still have a lot to learn about these metabolite transporters.

## The Solute Carrier (SLC) Family

Transport of substrates across biological membranes between and among organelles is an important feature of eukaryotic cells. The SLC family, the second largest family of membrane proteins, is a large group of membrane transport proteins; in humans, there are 456 known members that are grouped into 65 subfamilies (Höglund et al., 2011; Perland and Fredriksson, 2017). SLCs facilitate the movement of otherwise membrane-impermeable solutes—such as amino acids, ions, nucleotides, sugars and drugs—across biological membranes. The family includes functionally related proteins that mediate the transport and exchange of solutes across cell membranes. Transport can be facilitative by simply allowing solutes to equilibrate across a membrane according to their relative distribution on either side. Additionally, SLCs can mediate secondary active transport by coupling the downhill flow of one substrate, often an ion, to the uphill movement of another substrate against its relative gradient across a membrane. Primary active transporters, ion channels and aquaporins are not included in the SLC family. The criterion for membership in the SLC family is being an integral membrane protein that transports a solute. Not surprisingly, the SLC family is structurally quite diverse. However, within an individual sub-family, members often share more than 20% sequence homology (Hediger et al., 2004). Table 1 describes the current list of SLC family members based on http://slc.bioparadigms.org and provides references that review each subfamily. Families SLC53-65 are newly registered, and are based on a work presented at the BioMedical Transporters 2017 conference in Lausanne, Switzerland.

**Table 1:** Abridged list of current SLC families^a^.

SLC Subfamily	Description	Number of members	Reference
SLC1	High-affinity glutamate and neutral amino acid transporter family	7	Kanai and Hediger, 2003, 2004; Gegelashvili et al., 2006; Kanai et al., 2013; Nakagawa and Kaneko, 2013
SLC2	Facilitative GLUT transporter family	18 (including 4 pseudogene: SLC2A3P1, SLC2A3P2, SLC2A3P4, SLC2AXP1)	Uldry and Thorens, 2004; Simpson et al., 2008; Mueckler and Thorens, 2013; Hevia et al., 2015; Barron et al., 2016
SLC3	Heavy subunits of the heteromeric amino acid transporters	2	Palacín and Kanai, 2004; Verrey et al., 2004; Bergeron et al., 2008; Schweikhard and Ziegler, 2012; Fotiadis et al., 2013
SLC4	Bicarbonate transporter family	10 (SLC4A6 and SLC4A7 are the same)	Alper et al., 2001, 2002; Romero, 2005; Alper, 2006; Gill and Boron, 2006; Pushkin and Kurtz, 2006; Parker and Boron, 2013; Romero et al., 2013; Aalkjaer et al., 2014
SLC5	Sodium glucose cotransporter family	12	Bergeron et al., 2008; Wright, 2013
SLC6	Sodium- and chloride-dependent neurotransmitter transporter family	22 (1 pseudogene SLCA10P)	Pramod et al., 2013
SLC7	Cationic amino acid transporter/glycoprotein-associated family	15 (including 2 pseudogenes SLC7A5P1 and SLC7A15P)	Palacín and Kanai, 2004; Verrey et al., 2004; Bergeron et al., 2008; Schweikhard and Ziegler, 2012; Fotiadis et al., 2013
SLC8	Na^+^/Ca^2+^ exchanger family	4	Blaustein and Lederer, 1999; Quednau et al., 2004; DiPolo and Beaugé, 2006; Khananshvili, 2013
SLC9	Na^+^/H^+^ exchanger family	18 (including 5 pseudogenes)	Orlowski and Grinstein, 2004; Donowitz et al., 2013; Fuster and Alexander, 2014; Padan and Landau, 2016
SLC10	Sodium bile salt cotransport family	7	Geyer et al., 2006; Claro da Silva et al., 2013
SLC25	Mitochondrial carrier family	60 (including 7 pseudogenes)	Palmieri, 2004, 2013; Haitina et al., 2006; Clémençon et al., 2013; Palmieri and Monné, 2016
SLC53	Phosphate carriers	1	
SLC54	Mitochondrial pyruvate carriers	3	
SLC55	Mitochondrial cation/proton exchangers	3	
SLC56	Sideroflexins	5	
SLC57	Non-imprinted in Prader-Willi/Angelman syndrome chromosome region (NiPA) -like magnesium transporter family	6	
SLC58	MagT-like magnesium transporter family	2	
SLC59	Sodium-dependent lysophosphatidylcholine symporter family	2	
SLC60	Glucose transporters	2	
SLC61	Molybdate transporter family	1	
SLC62	Pyrophosphate transporters	1	
SLC63	Sphingosine-phosphate transporters	3	
SLC64	Golgi Ca2^+^/H^+^ exchangers	1	
SLC65	Niemann-Pick C (NPC)-type cholesterol transporters	2	


*^*a*^The SLC subfamily, description and numbers of members in each subfamily are shown. Further information on the SLC genes can be found at*http://slc.bioparadigms.org.

## Mitochondrial Carrier Family (SLC25)

The Solute Carrier 25 (SLC25) family transports solutes across the inner mitochondrial membrane (IMM), although several members of this family are localized to other cellular organelles such as chloroplasts and peroxisomes (Visser et al., 2002; Bedhomme et al., 2005). The MCF is the largest SLC subfamily and all members are encoded by the nuclear genome. As such, they are synthesized by cytoplasmic ribosomes and need to be imported from the cytosol to their final location. 35 members have been identified in yeast, 58 members in *Arabidopsis thaliana*, and 53 members have been identified in humans. As summarized in Table 2 (Palmieri and Pierri, 2010), substrates for approximately one third of the MCF have yet to be identified. Tissue distribution can vary from ubiquitous [e.g., SLC25A6 (Stepien et al., 1992)] to tissue-specific [e.g., SLC25A31 (Dolce et al., 2005; Rodić et al., 2005)].

**Table 2:** Current list of MCF members^a^.

SLC name	Human protein name	Substrates	Yeast orthologs^b^
SLC25A1	CIC (citrate carrier)	Citrate, isocitrate, malate, phosphoenolpyruvate (PEP)	CTP1
SLC25A2	ORC2 (ornithine carrier 2)	ornithine, citrulline, lysine, arginine, histidine	ORT1
SLC25A3	PHC (phosphate carrier)	Phosphate, Cu^++^	MIR1, PIC2
SLC25A4	ANT1 (adenine nucleotide translocase-1)	ADP, ATP	AAC3, AAC1, AAC2
SLC25A5	ANT2 (adenine nucleotide translocase-2)	ADP, ATP	AAC3, AAC1, AAC2
SLC25A5P1	Pseudogene		
SLC25A6	ANT3 (adenine nucleotide translocase-3)	ADP, ATP	AAC3, AAC1, AAC2
SLC25A6P1	Pseudogene		
SLC25A7	UCP1 (uncoupling protein 1)	H^+^	
SLC25A8	UCP2 (uncoupling protein 2)	four-carbon metabolites (C4)*^c^* H^+^	
SLC25A9	UCP3 (uncoupling protein 3)	H^+^	
SLC25A10	DIC (dicarboxylate carrier)	Malate, phosphate, succinate, sulfate, thiosulphate	DIC1
SLC25A11	OGC (oxoglutarate carrier)	2-oxoglutarate, malate	DIC1
SLC25A12	AGC1 (aspartate/glutamate carrier 1)	Aspartate, glutamate	AGC1
SLC25A13	AGC2 (aspartate/glutamate carrier 2)	Aspartate, glutamate	AGC1
SLC25A14	UCP5 (uncoupling protein 5)	***Orphan***	DIC1
SLC25A15	ORC1 (ornithine carrier 1)	Ornithine, citrulline, lysine, arginine	ORT1
SLC25A15P1	Pseudogene		
SLC25A16	GDC (Graves’ disease carrier)	***Orphan***	LEU5, YPR011C
SLC25A17	Peroxisomal membrane protein PMP34	CoA, FAD, NAD+, AMP, ADP, PAP, dPCoA, FMN	ANT1
SLC25A18	GC2 (glutamate carrier 2)	Glutamate	AGC1
SLC25A19	DNC (deoxynucleotide carrier)*^d^*	thiamine pyrophosphate, thiamine monophosphate, (deoxy)nucleotides	TPC1
SLC25A20	CAC (carnitine/acylcarnitine carrier)	Carnitine, acylcarnitine	CRC1
SLC25A20P1	Pseudogene		
SLC25A21	ODC (oxoadipate carrier)	Oxoadipate, oxoglutarate	ODC1, ODC2
SLC25A22	GC1 (glutamate carrier 1)	Glutamate	AGC1
SLC25A23	Calcium-binding mitochondrial carrier protein SCaMC-3	ATP-Mg2^+^, ATP, ADP, AMP, Pi	SAL1
SLC25A24	Calcium-binding mitochondrial carrier protein SCaMC-1	ATP-Mg^2+^, ATP, ADP, AMP, Pi	SAL1
SLC25A25	Calcium-binding mitochondrial carrier protein SCaMC-2	ATP-Mg^2+^ *^e^*	SAL1
SLC25A26	*S*-adenosylmethionine mitochondrial carrier protein (SAMC)	*S*-adenosyl-methionine, *S*-adenosyl-homocysteine	SAM5
SLC25A27	UCP4 (uncoupling protein 4)	***Orphan***	
SLC25A28	Mitoferrin 2 (Mfrn2)	Fe^2+^	MRS3, MRS4
SLC25A29	ORNT3	Ornithine, acylcarnitine	YMC2, YMC1
SLC25A30	Kidney mitochondrial carrier protein 1 or UCP6 (uncoupling protein 6)	***Orphan***	DIC1
SLC25A31	AAC4, ANT4 (adenine nucleotide carrier 4)	ADP, ATP	AAC2, AAC3
SLC25A32	MFT	Folate	FLX1, YIA6, YEA6
SLC25A33	PNC1 (pyrimidine nucleotide carrier 1)	UTP	RIM2
SLC25A34		***Orphan***	OAC1
SLC25A35		***Orphan***	OAC1
SLC25A36	PNC2 (pyrimidine nucleotide carrier 2)	Pyrimidine nucleotides	RIM2
SLC25A37	Mitoferrin 1 (Mfrn1)	Fe^2+^	MRS4, MRS3
SLC25A38		Glycine	HEM25
SLC25A39		***Orphan***	MTM1
SLC25A40		***Orphan***	MTM1
SLC25A41	SCaMC-3Like	ATP-Mg/Pi*^f^*	SAL1
SLC25A42	Mitochondrial coenzyme A transporter	CoA, ADP, ATP, adenosine 3′,5′-diphosphate, dPCoA	LEU5, YPR011C
SLC25A43		***Orphan***	
SLC25A44		***Orphan***	
SLC25A45		***Orphan***	YMC1, YMC2
SLC25A46		***Orphan***	
SLC25A47		***Orphan***	
SLC25A48		***Orphan***	YMC2, YMC1
SLC25A49	Mitochondrial carrier homolog (MTCH) 1	***Orphan***	
SLC25A50	MTCH2	***Orphan***	
SLC25A51	Mitochondrial carrier triple repeat protein (MCART) 1	***Orphan***	
SLC25A51P1	Pseudogene		
SLC25A51P2	Pseudogene		
SLC25A51P3	Pseudogene		
SLC25A52	MCART2	***Orphan***	
SLC25A53	MCART6	***Orphan***	


*^*a*^The SLC and protein names, and transported substrate(s) of each MCF member. Further information on the MCF can be found at http://slc.bioparadigms.org. ^*b*^More information about orthology can be found at Alliance of Genome Resources website. ^*c*^SLC25A8 is a transporter of C4 metabolites such as oxaloacetate, a function that matches well with its close phylogenetic relationship with SLC25A10 (Vozza et al., 2014). ^*d*^Contrary to its name, SLC25A19 is now recognized as a carrier for thiamine pyrophosphate and not for deoxynucleotides (Lindhurst et al., 2006; Kang and Samuels, 2008). ^*e*^SLC25A25 has been shown very recently to transport ATP-Mg^2+^ (Hofherr et al., 2018). ^*f*^SLC25A41 transports ATP-Mg/Pi in a calcium-independent manner (Traba et al., 2009)*.

**FIGURE 1:**
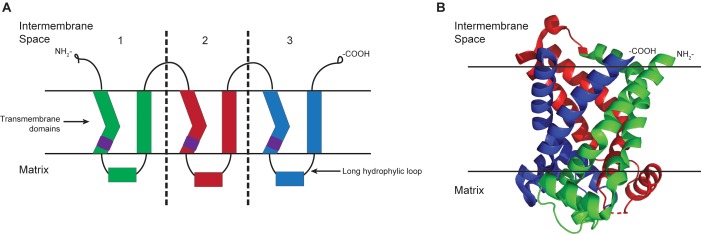
Schematic of the MCF tripartite structure. **(A)** The structure of members of the mitochondrial carrier family can be seen as three similar parts/domains with approximately 100 amino acids each. In each part, there are two alpha-helix transmembrane segments connected by a long matrix localized hydrophilic loop. The signature motif, PX(D/E)XX(K/R), indicated by purple cylinders, is at the C-terminus of the odd-numbered transmembrane helices. Both the NH2 and COOH termini are on the intermembrane space side of the mitochondrial inner membrane. **(B)** Structure of the yeast ADP/ATP carrier protein isoform 2 from protein data bank (entry 4C9G) is modified to show how the different domains are folded in a protein.

SLC25 members are generally characterized by the presence of a tripartite structure of approximately 300 amino acids, six conserved transmembrane regions, and the three-fold repeated MCF signature motif, P-X-[DE]-X-X-[RK] (Figure 1). Although their substrates vary in size and nature, most members catalyze the exchange of one solute for another (antiport), couple the transport of one solute with another (symport), or facilitate the transport of a solute (uniport). Because of their sequence similarity, it is assumed that the transport mechanism is similar for the extended family.

### Physiology of MCF

Collectively, the MCF transports a wide range of solutes across the IMM. In this capacity, they act as important bridges that link many biochemical pathways that are otherwise compartmentalized in either the cytosol or the mitochondrial matrix (Palmieri, 2014). Solutes transported include protons, nucleotides, amino acids, carboxylic acids, inorganic ions, and cofactors. Their fundamental role in enabling metabolic compartmentalization cannot be overemphasized. SLC25 family members are involved in metabolic pathways such as heme synthesis and metal homeostasis (SLC25A28, SLC25A37, and SLC25A38), fatty acid metabolism (SLC23A1 and SLC25A20), amino acid metabolism (SLC25A12, SLC25A13, SLC25A18, and SLC25A22), nucleic acid metabolism (SLC25A26, SLC25A33, and SLC25A36), urea production (SLC25A2, SLC25A13, and SLC25A15) (Shayakul and Hediger, 2004; Shayakul et al., 2013; LeMoine and Walsh, 2015), OXPHOS (SLC25A3, SLC25A4, SLC25A5, SLC25A6, and SLC25A31) and heat generation (SLC25A7 and SLC25A9). As a thorough discussion of the entire SLC family is not the goal of this review, in the next four sections, the physiology of select SLC25 members is briefly discussed to illustrate the diversity of cellular functions in which its members participate.

#### The ADP/ATP Carrier Protein Is Essential for OXPHOS

Eukaryotic cells make energy in the form of ATP in the mitochondrial matrix and the ATP is translocated through the impermeable IMM to power many processes in the cell. The ADP/ATP carriers (AACs) provide the means of transport of ATP and its precursor ADP, across the IMM. Under physiological conditions, 1 molecule of ADP from the cytosol is exchanged for 1 molecule of matrix-localized ATP by the activity of the ADP/ATP carrier. AAC, referred to as ANT in humans, is a notable MCF member as it was the first to have its amino acid sequenced (Aquila et al., 1982) and its 3D structure solved (Pebay-Peyroula et al., 2003). Similar to all members of the MCF and regarded as a paradigm for this family, AACs are nuclear-encoded, integral membrane proteins with approximately 300 amino acids arranged into three repeats linked by two loops on the cytosolic side. There are two transmembrane α-helices in each repeat connected together by a long loop on the matrix side, giving the carrier a threefold pseudosymmetry (Palmieri, 2013).

One of the most abundant proteins in the IMM, Aacs are encoded by multiple different genes in both unicellular and multicellular eukaryotes. There are three yeast AAC isoforms and four human ANT isoforms. The human ANT isoforms overlap in their expression pattern but exhibit tissue-specificity. ANT1 (SLC25A4) is the most equivalent to yeast Aac2p and the predominant isoform in the heart and skeletal muscle (Stepien et al., 1992). *ANT2* (*SLC25A5*) is mostly expressed in regenerative tissues such as the kidney and liver, *ANT3* (*SLC25A6*) is ubiquitously expressed at low baseline levels, and *ANT4* (*SLC25A31*) is selectively expressed in the testis (Stepien et al., 1992; Doerner et al., 1997; Dolce et al., 2005; Rodić et al., 2005; Kim et al., 2007; Dupont and Stepien, 2011). Aac2p is the most abundant of all three isoforms in yeast and the only one absolutely required for OXPHOS and growth on respiratory carbon sources (Lawson et al., 1990). Aac1p and Aac3p are minor isoforms in yeast that are undetectable at the protein level under normal growth conditions. Aac1p expression is repressed in hypoxic conditions (Gavurníková et al., 1996) and Aac3p expression is induced in anaerobic situations (Sabová et al., 1993).

Originally thought to consist of individual complexes in a functional chain, the advent of Blue-Native Polyacrylamide Gel-Electrophoresis (BN-PAGE) (Schägger and von Jagow, 1991), a gentle electrophoretic technique for the analysis of protein–protein interactions, facilitated the discovery that the respiratory complexes interact to form higher-order supramolecular assemblies of varying stoichiometry termed respiratory supercomplexes (RSCs) (Cruciat et al., 2000; Schägger and Pfeiffer, 2000; Acín-Pérez et al., 2008; Moreno-Lastres et al., 2012; Gu et al., 2016; Letts et al., 2016; Wu et al., 2016). In yeast which lack complex I, RSCs are composed of complexes III and IV whereas in mammals, RSCs consist of complexes I, III, and IV (Schägger and Pfeiffer, 2000). Thus, RSCs are an evolutionarily conserved organizing principle of the electron transport chain. Recently it was demonstrated that RSCs are functional entities (Barrientos and Ugalde, 2013; Lapuente-Brun et al., 2013) whose structures have since provided novel insight into the potential benefits that they may confer (Schäfer et al., 2007; Althoff et al., 2011; Dudkina et al., 2011; Genova and Lenaz, 2014; Gu et al., 2016; Letts et al., 2016; Wu et al., 2016). Functional benefits of RSCs that have been suggested but not yet proven include: improved electron transfer efficiency and reduced ROS generation, each stemming from a substrate channeling based mechanism; increased metabolic flexibility resulting from changes in RSC composition; and finally, enhanced stability and functionality of all participating complexes in the specific context of the protein-dense IMM (Barrientos and Ugalde, 2013; Milenkovic et al., 2017). Nevertheless, there is still some controversy as to the functional and physiological relevance of RSCs.

About 10 years ago, a new functional entity was shown to interact with yeast RSCs: Aac2p (Claypool et al., 2008; Dienhart and Stuart, 2008). More recently, this association was shown to be evolutionarily conserved as two distinct human ANT isoforms also form complexes with RSCs (Lu et al., 2017). Functionally, this conserved interaction could benefit both RSCs and the AACs. Specifically, the electrogenic exchange of ATP_in_/ADP_out_ by AAC/ANT is positively influenced by the membrane potential (ΔΨ) across the IMM (Krämer and Klingenberg, 1980) which of course is established by the electron transport chain. Similarly, by dissipating the electrical gradient, productive AAC/ANT transport makes it easier for RSCs to pump protons. As such, it is reasonable to hypothesize that this known functional synergy is further enhanced by being physically associated.

The absence of Aac2p in yeast impairs OXPHOS (Lawson et al., 1990; Heidkämper et al., 1996; Muller et al., 1996; Fontanesi et al., 2004; Claypool et al., 2008; Dienhart and Stuart, 2008). Prior mutagenic studies of Aac2p suggested that COX (complex IV) function is dependent on Aac2p function or expression (Muller et al., 1996; Müller et al., 1997). More recently, several groups demonstrated that there is a specific reduction in complex IV activity in yeast strains lacking Aac2p (Heidkämper et al., 1996; Muller et al., 1996; Fontanesi et al., 2004; Claypool et al., 2008; Dienhart and Stuart, 2008). These observations suggest that Aac2p nucleotide transport activity and/or its interaction with the RSCs are critical determinants of optimal COX activity.

ADP/ATP carriers interact with RSCs in the presence of cardiolipin, a unique phospholipid found exclusively in the mitochondrion (Claypool et al., 2008). Available structures of AAC/ANTs depict three tightly bound cardiolipin molecules per monomer (Beyer and Klingenberg, 1985; Pebay-Peyroula et al., 2003; Ruprecht et al., 2014). In the absence of cardiolipin, Aac2p function is impaired and Aac2p assembly is drastically altered (Jiang et al., 2000; Claypool et al., 2008). The absence of cardiolipin also destabilizes the RSCs (Zhang et al., 2002; Pfeiffer et al., 2003), including its association with Aac2p (Claypool et al., 2008). That Aac2p assembly and function is cardiolipin-dependent has led to the hypothesis that the assembly and function of AAC/ANTs may be the “Achilles heel” of a multitude of cardiolipin-based diseases (Klingenberg, 2008; Claypool, 2009). The structural changes in RSCs and Aac2p that occur in the absence of cardiolipin have clear functional consequences (Claypool et al., 2008). However, the relative contribution of each structural change— impaired assembly of RSCs, Aac2p, or RSC-Aac2p— that occurs in the absence of cardiolipin to the associated mitochondrial dysfunction has not been established.

It has also been hotly debated whether the protein exists and/or functions as a monomer or dimer. Mitochondrial carriers were originally accepted to exist and function as homo-dimers (Lin et al., 1980; Klingenberg, 1981; Palmisano et al., 1998; Schroers et al., 1998; Kotaria et al., 1999; Trézéguet et al., 2000; Capobianco et al., 2002; Dyall et al., 2003; Nury et al., 2005; Postis et al., 2005). A number of studies, motivated by the crystal structures (Kunji and Harding, 2003; Pebay-Peyroula et al., 2003; Ruprecht et al., 2014), have challenged this initial view and instead provided evidence that AACs function, and in fact exist in the IMM, as monomers (Kunji and Harding, 2003; Bamber et al., 2006, 2007a,b; Kunji and Crichton, 2010). Obviously, AACs cannot be monomeric in the IMM and interact with RSCs in a cardiolipin-dependent manner. Additional work is needed to reconcile these very different models of AAC connectivity.

#### Mitoferrins Are Fundamental to Iron Transport

**FIGURE 2:**
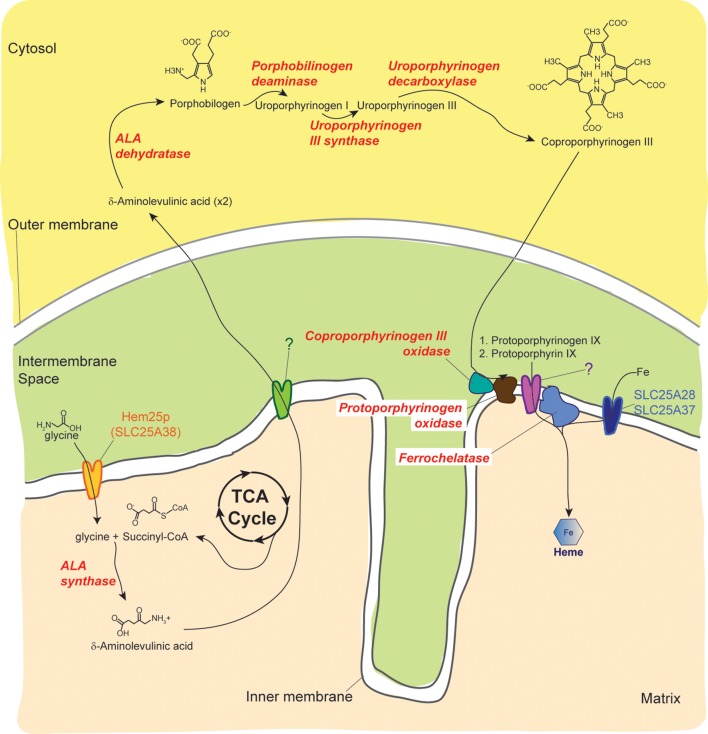
Overview of the heme biosynthetic pathway. Three known MCF members are involved in heme biosynthesis. Following its transport into the matrix by Hem25p/SLC25A38, glycine is condensed with succinyl-CoA by ALA synthase to form δ-aminolevulinic acid. The next four steps of the heme biosynthetic pathway occur in the cytosol. The identity of the protein, which may be a MCF member, that mediates the transport of δ-aminolevulinic acid across the inner membrane has not been determined. The active sites of coproporphyrinogen III oxidase and Protoporphyrinogen oxidase face the intermembrane space. In contrast, the final step in the heme biosynthetic pathway occurs in the matrix and is catalyzed by ferrochelatase. The identity of the protein, which may be a MCF member, that transports protoporphyrin IX to the matrix has not been determined. Ferrochelatase incorporates iron (Fe), transported into the matrix by the mitoferrins SLC25A28 and SLC25A37, into protoporphyrin IX to produce heme.

Iron is essential for mitochondrial function (Levi and Rovida, 2009). Mitochondria themselves are intimately involved in the regulation of cellular iron. Iron is important in the heme biosynthetic pathway in the reaction step of ferrous iron incorporation into protoporphyrin IX catalyzed by ferrochelatase (Figure 2) (Ponka, 1997). Heme is needed for synthesis of the mitochondrial cytochromes which are electron carriers critical for OXPHOS. In addition, iron-sulfur cluster biogenesis occurs in the mitochondrial matrix and is tightly linked to many other cellular processes such as heme biosynthesis, ribosome assembly, DNA synthesis, and translation initiation (Lill and Mühlenhoff, 2008; Lill, 2009). *SLC25A28* and *SLC25A37* encode Mitoferrin 2 (MFRN2) and Mitoferrin 1 (MFRN1), respectively, which are involved in iron import into the mitochondrion. In zebrafish and mammals, MFRN1 is expressed predominantly in hematopoietic tissues whereas MFRN2, with 65% amino acid identity to its paralog, is widely expressed (Shaw et al., 2006; Amigo et al., 2011). MFRN2 has about 38% identity to Mrs3p and Mrs4p (Shaw et al., 2006), two yeast transporters originally identified as suppressors of an intron splicing defect (Waldherr et al., 1993) that have since been associated with iron transport (Foury and Roganti, 2002). Yeast lacking Mrs3p and Mrs4p exhibit poor growth in iron-depleted conditions (Foury and Roganti, 2002). *MFRN1* loss-of-function in mice and zebrafish results in reduced iron uptake into mitochondria and defective hemoglobin synthesis (Shaw et al., 2006). In non-erythroid cells, MFRN2 and MFRN1 are both involved in mitochondrial iron uptake (Paradkar et al., 2009). When both transporters are silenced in non-erythroid cells, heme synthesis is severely compromised; further overexpression of one can functionally compensate for the loss of the other (Paradkar et al., 2009). These results establish the fundamental importance of these proteins in mitochondrial iron metabolism in erythroid and non-erythroid cells.

#### Uncoupling Proteins Provide a Pathway for Proton Leakage

**FIGURE 3:**
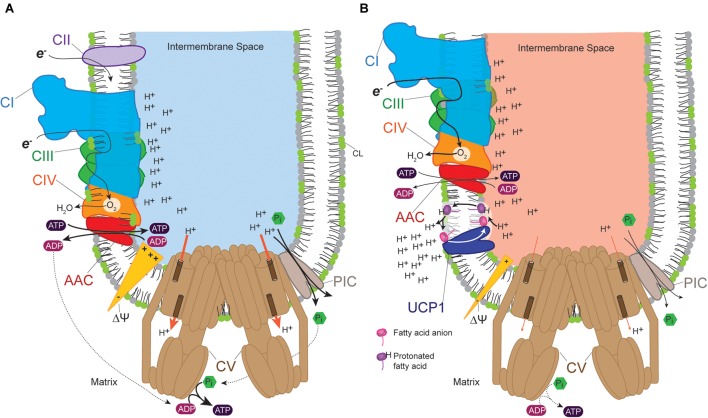
Uncoupling protein (UCP) provides a pathway for proton leak. **(A)** Proton pumping across the inner membrane into the intermembrane space by complexes I, III, and IV occurs as electrons (*e*^-^) flow down the transport chain. This generates a proton-based electrochemical gradient (Δψ) that is consumed by the ATP synthase to produce ATP, thus coupling ATP synthesis to proton pumping. The proton gradient also provides the driving force for the exchange of ADP and ATP by AAC and the uptake of *P*_i_ by PIC, transport processes that are essential for OXPHOS. **(B)** UCPs provide an alternative means to equilibrate the proton gradient which effectively uncouples proton pumping from ATP synthesis. This results in increased flux of electrons through the electron transport chain which generates heat (indicated by *red* intermembrane space). In **(A,B)**, line thickness reflects relative flux/activity.

The UCPs are regulated mitochondrial proteins known to transport protons, anions or other mitochondrial substrates (Jezek et al., 2010; Fedorenko et al., 2012; Porter, 2012; Monné et al., 2018). Six UCP homologs have been discovered in humans—UCP1 or thermogenin (Heaton et al., 1978), UCP2 (Fleury et al., 1997), UCP3 (Boss et al., 1997), UCP4 (Mao et al., 1999), UCP5 or BMCP1 for brain mitochondrial carrier protein 1 (Sanchis et al., 1998), and UCP6 or KMCP1 for kidney mitochondrial carrier protein 1 (Haguenauer et al., 2005). UCPs uncouple OXPHOS from ATP synthesis; they dissipate proton gradients by allowing protons that have been pumped into the intermembrane space by respiratory complexes to flow back into the mitochondrial matrix without being utilized for ATP synthesis (Figure 3). The translocation of hydrogen ion by UCPs requires fatty acids and this activity is inhibited by purine nucleotides such as GDP. Until recently, the role of fatty acids in activating the uncoupling process was hotly debated in relation to the putative UCP transport mechanism. Proton and anion transport by UCP involves fatty acids, as extensively reviewed (Jezek et al., 2018). According to the fatty acid cycling mechanism, protonated free fatty acids spontaneously flip to the matrix IMM leaflet where they release protons based on the proton gradient across the IMM. In turn, UCPs provide a conduit consisting of basic amino acids that facilitates the movement of the FA anion back to the IMS leaflet, a process that is also driven by the electrochemical gradient. In the net, these processes dissipate the proton gradient (Kamp and Hamilton, 1992; Kamp et al., 1993, 1995; Jezek et al., 1997a,b; Jabùrek et al., 2001; Jezek et al., 2010, 2018). This model is validated by nuclear magnetic resonance and functional mutagenesis studies on UCP2 which provided molecular and structural support for this protonophoretic model (Berardi and Chou, 2014).

The UCP1 is thought to be exclusively found in brown adipose tissue and is the only UCP responsible for adaptive adrenergic non-shivering thermogenesis (Nicholls et al., 1978; Matthias et al., 2000; Golozoubova et al., 2001; Porter, 2008). As such, it is firmly established that UCP1 functions as a true UCP that utilizes the electrochemical gradient generated by the respiratory chain to produce heat instead of ATP. Also, there seems to be a close relationship between mitochondrial ROS and UCP1-dependent thermogenesis although whether or not superoxide modulates UCP1 function is debated (Echtay et al., 2002; Silva et al., 2005; Chouchani et al., 2017). Nonetheless, work with *ucp1* knockout (*ucp1*^-/-^) mice has established that UCP1 is a target of redox modification *in vivo* (Chouchani et al., 2016). A recent study showed that brown adipose tissue (BAT) from *ucp1*^-/-^ mice have reduced respiratory chain proteins and increased host defense signaling following exposure to cold (Kazak et al., 2017). Intriguingly, BAT-derived mitochondria from *ucp1*^-/-^ mice are more sensitive to calcium overload in a ROS-dependent manner (Kazak et al., 2017). Thus, though UCP1 is traditionally linked to thermogenesis, it is becoming clear that UCP1 function extends beyond thermogenesis.

UCP2-5 are not involved in thermogenesis even though they provide mild uncoupling which may be protective against oxidative stress (Jezek et al., 2018). UCP2 transports C4 metabolites such as oxaloacetate, a function that matches well with its close phylogenetic relationship with the dicarboxylic acid carrier, SLC25A10 (Vozza et al., 2014). It is widely expressed (Fleury et al., 1997; Gimeno et al., 1997) and has numerous pathophysiological roles. For instance, due to its ability to reduce ROS generation, UCP2 participates in both host immunity and the inflammatory response (Nègre-Salvayre et al., 1997; Arsenijevic et al., 2000; Mattiasson and Sullivan, 2006). In addition, UCP2 has been implicated in body mass regulation, glucose metabolism, and carcinogenesis (Zhang et al., 2001; Horimoto et al., 2004; Mattiasson and Sullivan, 2006; Derdak et al., 2008; Li et al., 2013, 2015; Vozza et al., 2014; Sreedhar et al., 2017). UCP3 is expressed mainly in the skeletal muscle and brown adipose tissue, and minimally in the heart (Boss et al., 1997; Vidal-Puig et al., 1997) where it is important for ROS attenuation but not body mass regulation or fatty acid metabolism (Vidal-Puig et al., 2000). Indeed, mitochondria from mice with lower levels of UCP3 have increased ROS production and oxidative damage further suggesting that UCP3 protects against ROS and oxidative damage (Brand et al., 2002). UCP4 is predominantly expressed in the nervous system including different regions of the brain, the spinal cord, hair cells of the inner ear, and Merkel cells in the skin (Liu et al., 2006; Smorodchenko et al., 2009, 2011). UCP4 overexpressing neuronal cell lines have reduced OXPHOS with a corresponding increase in glucose uptake and glycolysis (Liu et al., 2006). These metabolic changes correlate with a drop in ROS production, a reduced tendency for calcium overload and an overall increased resistance to apoptosis (Liu et al., 2006). Overexpression of UCP4 in pre-adipocyte cell lines stimulates proliferation, inhibits differentiation into adipocytes and protects against apoptosis (Zhang et al., 2006). Furthermore, impaired insulin sensitivity and mitochondrial biogenesis, decreased mtDNA level and increased ROS production occurs in adipocyte cell lines overexpressing UCP4 suggesting a global negative impact of UCP4 on mitochondrial function (Gao et al., 2010). However, in UCP4 overexpressing L6 myocytes, insulin sensitivity is improved with no change in intracellular ROS production, mtDNA levels or mitochondrial biogenesis (Gao et al., 2011). Regulated UCP4 expression, therefore, seems to be critical for optimal mitochondrial and cellular function.

UCP5 is expressed principally in the central nervous system and has three different forms (long form, UCP5L with 325 amino acids; short form, UCP5S with 322 amino acids; and short insert form, UCP5S1 with 353 amino acids) (Ramsden et al., 2012). UCP5 overexpression in human SH-SY5Y cells increases proton leak, reduces mitochondrial membrane potential and ATP production, and increases oxygen consumption (Kwok et al., 2010). UCP6 has not been well studied. To date, it is associated with carcinogenesis (Nohara et al., 2012) and its expression in the kidney cortex is increased following pro-oxidant states (Haguenauer et al., 2005).

#### The Citrate Carrier Has a Pervasive Role in Cellular Metabolism

**FIGURE 4:**
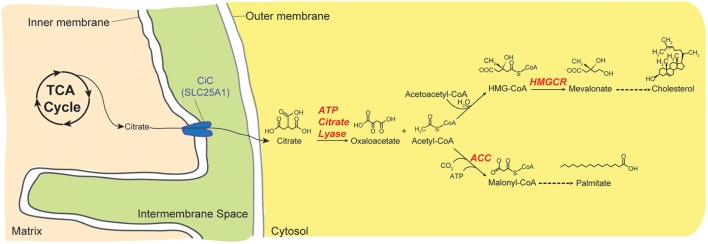
Citrate provides substrates for anabolic pathways. Citrate is a substrate for many biological reactions. Here, the role of citrate in fatty acid and cholesterol synthesis is depicted. Citrate is transported from the mitochondrion into the cytoplasm by CiC/SLC25A1. Once in the cytoplasm, it is broken down into oxaloacetate and acetyl-CoA by the action of ATP Citrate Lyase. For fatty acid synthesis, acetyl-CoA is carboxylated by Acetyl-CoA Carboxylase (ACC) to malonyl-CoA. Alternatively, acetyl-CoA can be condensed with itself to generate acetoacetyl-CoA, which then reacts with another acetyl-CoA and water to form 3-hydroxy-3-methyl-glutaryl-CoA (HMG-CoA). Conversion of HMG-CoA to mevalonate, the committed step in cholesterol biosynthesis, is performed by 3-hydroxy-3-methyl-glutaryl-coenzyme A reductase (HMGCR).

The ubiquitously expressed mitochondrial CiC, also known as the tricarboxylate carrier, citrate transport protein or dicarboxylic acid transporter, is encoded by *SLC25A1* in mammals. It catalyzes the obligate electroneutral exchange of a tricarboxylate such as citrate for another tricarboxylate, a dicarboxylate such as malate, or phosphoenolpyruvate (Klingenberg, 1972; Bisaccia et al., 1989; Gnoni et al., 2009). CiC exports matricial citrate, the first product of the citric acid (Kreb; tricarboxylic acid) cycle, in exchange for cytosolic malate. Citrate is an important regulatory substrate for many metabolic reactions. As such, CiC provides substrates for both fatty acid and cholesterol biosynthesis (Figure 4) and helps in the transfer of reducing equivalents across the IMM (Palmieri et al., 2015). For fatty acid synthesis, citrate is source of carbons and also allosterically regulates the rate-limiting enzyme of this biosynthetic pathway. Cytoplasmic citrate is first cleaved into acetyl-CoA and oxaloacetate by ATP citrate lyase. Acetyl-CoA is then carboxylated into malonyl CoA by acetyl CoA carboxylase, the rate-limiting step in fatty acid synthesis. For cholesterol synthesis, two molecules of acetyl-CoA (also from Citrate by ATP citrate lyase) are condensed to yield acetoacetyl-CoA which is then condensed with another acetyl-CoA to yield 3-hydroxy-3-methylglutaryl-CoA (HMG-CoA). The committed step in cholesterol synthesis is the reduction of cytosolic HMG-CoA to mevalonate. Fatty acid and cholesterol synthesis also requires NADPH and some of these are generated from cytosolic oxaloacetate via malate dehydrogenase and malic enzyme (Palmieri, 2004). Citrate transport from the mitochondrion increases NADPH synthesis and the activity of human CiC is modulated by acetylation in activated immune cells (Palmieri et al., 2015). The orthologous yeast citrate transport protein Ctp1p, preferentially transports tricarboxylates versus other substrates, in contrast to the mammalian transporter which transports tricarboxylates, malate, and phosphoenolpyruvate to similar extents (Kaplan et al., 1995). By supplying citrate-derived acetyl-CoA that is required for normal nucleosome histone acetylation, SLC25A1 and its *Drosophila* ortholog, *scheggia (sea)* are also important for the maintenance of genome integrity (Morciano et al., 2009). Recently, it was shown that SLC25A1 is critical for tumor cell metabolism; it enables stem-like properties in cells, contributes to treatment resistance in cancer cells (Fernandez et al., 2018), and has been proposed as a metabolic oncogene (Kolukula et al., 2014). In another recent work, SLC25A1 and its genetic and functional interactions with SLC25A4 is required for optimal neuronal development, function and behavior (Gokhale et al., 2018). Therefore, CiC is critical for intermediary metabolism and has emerging roles in human development and disease including cancer.

### Pathology of MCF

The MCF provides substrates for various biochemical processes in the cell. Consistent with their diverse and fundamental roles in metabolism, the absence or dysfunction of assorted MCF members causes a wide variety of disorders including hematologic, neurologic, and cardiac diseases. Underlying many of these disorders is a defect in OXPHOS leading to disturbed mitochondrial energy metabolism that manifests in a wide variety of clinical signs and symptoms. A number of systemic diseases caused by mutations in genes encoding SLC25 members are discussed briefly next and Table 3 displays a summary of MCF-linked diseases clustered by presentation.

**Table 3:** Summary of known MCF-involved diseases clustered by systemic presentation.

System	Clinical presentation/disease and MCF member associated
Hematopoietic	**Sideroblastic anemia:** SLC25A38 OMIM 205950 (Guernsey et al., 2009; Harigae and Furuyama, 2010; Horvathova et al., 2010)
Metabolic	**Lactic acidosis:** SLC25A4 (Bakker et al., 1993; Palmieri et al., 2005; Thompson et al., 2016); SLC25A26 OMIM 616794 (Kishita et al., 2015); SLC25A42 (Almannai et al., 2018)
	**Citrullinemia:** SLC25A13 OMIM 603471 and 605814 (Kobayashi et al., 1999; Yasuda et al., 2000; Ohura et al., 2001; Tazawa et al., 2001; Fiermonte et al., 2008)
	**Hydroxyglutaric aciduria:** SLC25A1 OMIM 615182 (Muntau et al., 2000; Edvardson et al., 2013; Nota et al., 2013)
	**Hyperornithinemia-Hyperammonemia-Homocitrullinuria (HHH) Syndrome:** SLC25A15 OMIM 603861 (Camacho et al., 1999; Tsujino et al., 2000; Miyamoto et al., 2001; Salvi et al., 2001; Debray et al., 2008; Tessa et al., 2009)
	**Hypoglycemia, Hyperammonemia:** SLC25A20 OMIM 212138 (Stanley et al., 1992; Pande et al., 1993; Iacobazzi et al., 2004)
	**Congenital Hyperinsulinism:** SLC25A8 (González-Barroso et al., 2008; Ferrara et al., 2017)
	**Hypertriglyceridemia:** linkage to SLC25A40 (Rosenthal et al., 2013)
	**Exercise intolerance:** SLC25A32 OMIM 616839 (Schiff et al., 2016; Hellebrekers et al., 2017)
Cardiovascular	**Hypertrophic cardiomyopathy:** SLC25A4 OMIM 615418 (Palmieri et al., 2005; Echaniz-Laguna et al., 2012; Körver-Keularts et al., 2015), OMIM 617184 (Thompson et al., 2016); SLC25A3 OMIM 610773 (Mayr et al., 2007; Bhoj et al., 2015); SLC25A20 OMIM 212138 (Stanley et al., 1992; Pande et al., 1993; Huizing et al., 1998; Iacobazzi et al., 2004; Nakase et al., 2007; Van De Parre et al., 2008; Dong et al., 2011, 2015)
Pulmonary	**Respiratory insufficiency:** SLC25A26 OMIM 616794 (Kishita et al., 2015)
Musculoskeletal	**Myopathy and Muscular atrophy-like disease:** SLC25A4 OMIM 615418 (Bakker et al., 1993; Palmieri et al., 2005; Echaniz-Laguna et al., 2012; Körver-Keularts et al., 2015), OMIM 617184 (Thompson et al., 2016); SLC25A3 OMIM 610773 (Mayr et al., 2007); SLC25A26 OMIM 616794 (Kishita et al., 2015); SLC25A32 (Hellebrekers et al., 2017); SLC25A21 (Boczonadi et al., 2018); SLC25A42 OMIM 610823 (Shamseldin et al., 2016; Almannai et al., 2018)
	**Progressive External Ophthalmoplegia:** SLC25A4 OMIM 609283 (Kaukonen et al., 2000; Napoli et al., 2001; Lamantea et al., 2002)
Neurological	**Epileptic encephalopathy:** SLC25A12 OMIM 612949 (Wibom et al., 2009; Falk et al., 2014); SLC25A22 OMIM 609304 (Molinari et al., 2005; Molinari et al., 2009; Poduri et al., 2013); SLC25A42 (Almannai et al., 2018)
	**Microcephaly and Neural Tube closure defects:** SLC25A19 OMIM 607196 (Rosenberg et al., 2002; Lindhurst et al., 2006) **Neuropathy:** Progressive polyneuropathy – SLC25A19 OMIM 613710 (Spiegel et al., 2009); Charcot-Marie-Tooth Disease – SLC25A46 OMIM 616505 (Abrams et al., 2015; Charlesworth et al., 2016; Janer et al., 2016; Wan et al., 2016)
	**Ataxia, Myoclonus, dysarthria:** SLC25A32 (Hellebrekers et al., 2017)
Gastrointestinal	**Cholestatic jaundice:** SLC25A13 OMIM 605814 (Ohura et al., 2001; Tazawa et al., 2001; Tamamori et al., 2002)
	**Hepatic Steatosis:** SLC25A13 OMIM 603471 (Komatsu et al., 2008)
General	**Progeroid syndrome:** SLC25A24 OMIM 612289 (Ehmke et al., 2017; Writzl et al., 2017)
	**Fingernail dysplasia:** SLC25A16 OMIM 139080 (Khan et al., 2018)
	**Familial Synpolydactyly:** SLC25A21 (Meyertholen et al., 2012)
	**Predisposition to Metastatic paragangliomas:** SLC25A11 (Buffet et al., 2018)


Mitochondrial carriers encoded by *SLC25A28*, *SLC25A37*, and *SLC25A38* are important for heme synthesis which requires cellular iron, glycine and succinyl-CoA (Figure 2). Since red blood cells are very sensitive to defects in heme synthesis, dysfunction in any of these mitochondrial carriers causes anemia (Xu et al., 2013). The erythroid specific SLC25A38, which based on its requirement for erythropoiesis was initially predicted to encode an amino acid carrier capable of transporting glycine, is now regarded as a bonafide glycine transporter (Guernsey et al., 2009; Fernández-Murray et al., 2016; Lunetti et al., 2016). Pathogenic mutations in *SLC25A38* and knockdown experiments in zebrafish implicate the carrier in the etiology of congenital sideroblastic anemia (Guernsey et al., 2009) (OMIM 610819). Mutations in the carrier are the second most common cause of inherited sideroblastic anemia and may account for about a fifth of all cases (Harigae and Furuyama, 2010; Horvathova et al., 2010). As previously discussed, *SLC25A28* and *SLC25A37*, which encode the mitoferrins, are critical for iron homeostasis. Although no human mutations have been described for these carriers, MFRN1 has been shown to be important for heme synthesis in erythroid cells (Shaw et al., 2006).

*SLC25A46* encodes a novel outer mitochondrial membrane protein that is widely expressed in the nervous system (Haitina et al., 2006), and mutated in numerous neurological diseases including optic atrophy spectrum disorder, Charcot-Marie-Tooth type 2, Leigh syndrome, progressive myoclonic ataxia, and lethal congenital pontocerebellar hypoplasia (Abrams et al., 2015; Wan et al., 2016; Terzenidou et al., 2017) (OMIM 610826). Insight into each of these neurological diseases is hampered by the fact that SLC25A46 is an orphan member of the MCF whose substrate(s) has not been defined (Palmieri and Monné, 2016). However, given its unusual localization to the outer mitochondrial membrane, whether SLC25A46 functions as a transporter or instead has novel activities that are unrelated to its SLC25 membership remains an open question. Indeed, SLC25A46 has been implicated in both mitochondrial dynamics and cristae morphology (Abrams et al., 2015; Janer et al., 2016; Steffen et al., 2017).

Mitochondrial energy production is high in cardiac tissue and it is unsurprising that many mitochondrial carriers have been associated with cardiac disease, manifested in most cases as hypertrophic cardiomyopathy. Mutations in *SLC25A4*, encoding ANT1, are responsible for both the autosomal dominant and recessive cardiomyopathic type mitochondrial DNA depletion syndromes (OMIM 617184 and 615418, respectively) (Palmieri et al., 2005; Echaniz-Laguna et al., 2012; Körver-Keularts et al., 2015; Thompson et al., 2016), in addition to autosomal dominant progressive external ophthalmoplegia (OMIM 609283) (Kaukonen et al., 2000; Napoli et al., 2001; Komaki et al., 2002; Siciliano et al., 2003). Mutations in *SLC25A3* that encodes the phosphate carrier cause either hypertrophic cardiomyopathy and impaired function of other organs such as skeletal muscle (Mayr et al., 2007; Mayr et al., 2011) or isolated cardiomyopathy (Bhoj et al., 2015). Interestingly, in the latter case (Bhoj et al., 2015), the mutations discovered in *SLC25A3* were a mix of a single nucleotide change and a stretch of indels, both of which could potentially impact the two mammalian isoforms of the protein (Bhoj et al., 2015; Seifert et al., 2015). By disrupting the Urea Cycle, mutations in *SLC25A13*, whose protein product mediates the electrogenic exchange of aspartate for glutamate (Palmieri et al., 2001), cause adult onset citrulinemia (Kobayashi et al., 1999; Yasuda et al., 2000; Ohura et al., 2001; Tazawa et al., 2001; Fiermonte et al., 2008). Carnitine-acylcarnitine translocase deficiency resulting from many different mutations in *SLC25A20* results in a multi-systemic disorder that includes cardiomyopathy as one of its clinical features (OMIM 212138) (Stanley et al., 1992; Pande et al., 1993; Iacobazzi et al., 2004). A genome-wide association study reported an association between *UCP5* gene variants and the formation of atherosclerotic plaques suggesting that UCP5 has a protective role against atherosclerosis (Dong et al., 2011). Furthermore, UCP5 expression is increased in embolic stroke and multiple infarction brain lesions probably due to upregulation brought about by chronic ischemic stress (Nakase et al., 2007). However, since UCP5, like SLC25A38 and SLC25A46, is an orphan MCF, the underlying pathogenic mechanism is very much unclear at this time.

### Challenges to MCF-Focused Research

Mitochondrial carriers, and SLCs in general, perform a central role in metabolism and their association with a myriad of diseases makes them attractive candidates for basic and translational research. However, it has been noted that this area of research has not grown in commensurate proportion to its size or the gold mine its study could potentially reap (Cesar-Razquin et al., 2015). Most of these carriers are yet to be fully characterized and many of them remain totally uncharacterized. A number of technical factors have hampered growth of research focused on the extended membership of the SLC family that of course includes the MCF. A huge technical hurdle is the systemic absence of validated antibodies specific to most of these proteins. Further, many of the available antibodies are too weak to detect endogenous proteins whose expression is likely low and in general, many of the available reagents have not been rigorously characterized and validated (e.g., absence of signal with appropriate negative controls such as knockout cells). Compounding issues is the fact that many SLC members appear to have low immunogenicity which likely stems from the fact that they are polytopic membrane proteins that often display high interspecies conservation. Finally, transport assays are tedious to perform and limited by the volume of substrates available and/or required to de-orphanize a SLC protein.

Interestingly, a number of carriers have been shown to display substrate promiscuity by transporting more than one type of solute (Fiermonte et al., 2009). Two members in *Arabidopsis thaliana*, AtUCP1 and AtUCP2, previously thought to be UCPs and therefore named as such were recently assigned the function of transporting amino acids, dicarboxylates, phosphate, sulfate, and thiosulfate (Monné et al., 2018). The Pi carrier in mammals, SLC25A3, was originally described as a phosphate symporter (Wohlrab and Flowers, 1982; Seifert et al., 2015) whose mutation is associated with fatal childhood diseases (Mayr et al., 2007; Bhoj et al., 2015). Pic2p, originally thought to be a second albeit minor Pi carrier in yeast, actually functions as a Cu^++^ transporter responsible for the import of copper, required for COX assembly, into the mitochondrial matrix (Vest et al., 2013). Similarly, SLC25A3, which shares 65% similarity with Pic2p, was recently shown to have a conserved role for copper transport *in vivo* and *in vitro* (Vest et al., 2013; Boulet et al., 2017). Together, these results indicate that even well-established MCF members may have the capacity to transport additional presently unappreciated substrates.

Apparent functional redundancy is another recurring feature of mitochondrial carriers (Taylor, 2017). For example, mitochondrial nucleotide homeostasis not only involves the ADP/ATP carriers (SLC25A4, SLC25A5, SLC25A6, and SLC25A31) but also is influenced by the ATP-Mg/Pi carriers (SLC25A23, SLC25A24, SLC25A25, and SLC25A41). Indeed, in yeast, the calcium-dependent ATP-Mg/Pi carrier, Sal1p, can compensate for the absence of the major isoform of the ADP/ATP carrier, Aac2p, and provide a pool of mitochondrial ATP that is required for yeast viability (Chen, 2004; Cavero et al., 2005; Traba et al., 2008; Laco et al., 2010). Still, there are a lot of unknowns regarding how all of these different transporters maintain the nucleotide pool across the IMM. It has been suggested that different mammalian ADP/ATP carrier isoforms, which do not transport AMP, may have different preferred transport modes, i.e., ATP vs. ADP. For instance, based on its high expression in cancer cells, SLC25A5 (ANT2) was postulated to preferentially import ATP made by glycolysis, an activity that maintains the mitochondrial membrane potential and by extension, other essential mitochondrial functions (Stepien et al., 1992; Giraud et al., 1998; Chevrollier et al., 2005). However, it was recently demonstrated in a range of cancer cells that the uptake of ATP is in fact completely independent of the activity of any ADP/ATP carrier isoform (Maldonado et al., 2016). As such, it remains unresolved whether the different ANT isoforms do or do not have distinct transport activities and/or substrate preferences. Also unclear is how the calcium-dependent ATP-Mg/Pi carriers, which were originally thought to preferentially transport ATP but since demonstrated to also transport other adenine nucleotides (Fiermonte et al., 2004), interface with the ADP/ATP carriers to modulate mitochondrial nucleotide homeostasis. The requirement for calcium provides an opportunity for the flux of adenine nucleotides across the IMM to be regulated. Indeed, the calcium-dependent mitochondrial uptake of adenine nucleotides by SLC25A23 is required for the glucagon-stimulated increase in OXPHOS in liver mitochondria (Amigo et al., 2013). As research progresses in this area, identification of the substrate(s) transported by many of these proteins will provide foundational information as to their physiological roles.

## Mitochondrial Translation

The vast majority of mitochondrial proteins are translated in the cytosol and imported thereafter into the mitochondrion. For those proteins encoded by the mitochondrial genome (mtDNA), mitochondria have retained a dedicated, dual-origin translational machinery whose architecture is similar to that of bacteria. Consistent with this bacterial origin, mitochondrial translation is pharmacologically unaffected by cycloheximide, an inhibitor of cytosolic translation, and is instead sensitive to antibiotics such as puromycin and chloramphenicol. Eight polypeptides in yeast and thirteen polypeptides in humans are encoded by mtDNA and thus produced via mitochondrial translation. Apart from genes for these polypeptides, the mtDNA also encodes a set of transfer (t)RNAs, ribosomal (r) RNAs and in yeast, the RNA component of the mitochondrial RNAse P (Towpik, 2005; Walker and Engelke, 2008). The mitochondrial translation cycle is subdivided into four steps— initiation, elongation, termination and recycling—and nuclear encoded polypeptide factors are required at different steps for optimal mitochondrial translation (Towpik, 2005; Smits et al., 2010; Kehrein et al., 2013). An example of a nuclear-encoded factor in yeast is the mitochondrial translation initiation factor 3 (mIF3p), the *Saccharomyces cerevisiae* homolog of the bacterial translation initiation factor 3 (IF3). Its function is conserved and overlaps with human mIF3p (Atkinson et al., 2012; Kuzmenko et al., 2014), and its absence in yeast disrupts mitochondrial translation (Kuzmenko et al., 2016). In addition, a number of translational activators directly interacting with mRNAs of mitochondrial encoded polypeptides are necessary to optimize the translation of a specific mtDNA-encoded polypeptide, e.g., synthesis of Cox1p is affected when Mss51p is absent or limiting (Siep et al., 2000; Perez-Martinez et al., 2003, 2009; Barrientos et al., 2004; Fontanesi et al., 2011).

Translation of some mitochondrial proteins is tightly coupled to their assembly into respiratory complexes in a manner similar to a mechanism described as “controlled by epistasy of synthesis” (CES) that exists in the biogenesis of photosynthetic protein complexes (Choquet et al., 2001; Towpik, 2005). For example, in yeast, Cox1p synthesis is tightly coupled to the assembly of respiratory complex IV which helps to balance the production of subunits with their assembly into the holoenzyme which in the net preserves mitochondrial proteostasis (Perez-Martinez et al., 2003; Barrientos et al., 2004; Towpik, 2005; Soto et al., 2012).

## Emerging Roles in the Biogenesis of Cytochrome *C* Oxidase

**FIGURE 5:**
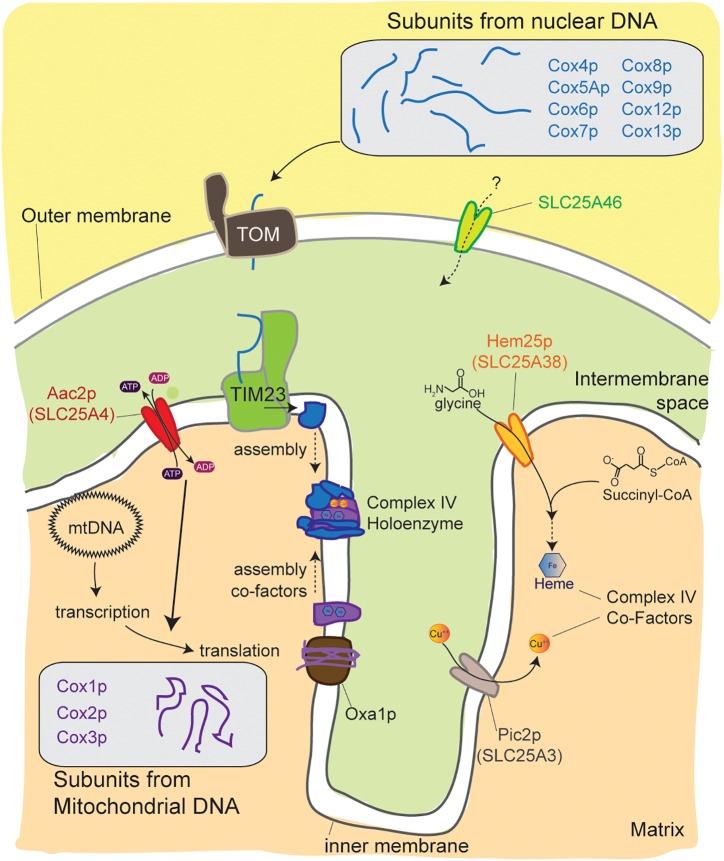
An emerging link between mitochondrial carriers and complex IV biogenesis. Cytochrome *c* oxidase is assembled from protein subunits that originate from the mitochondrial and nuclear genomes. Mitochondrial carriers support these processes at multiple levels. Mutations in the metazoan-specific SLC25A46, an unusual family member that resides in the outer membrane, decreases complex IV levels by an unclear mechanism. The absence of Aac2p function impairs translation of subunits encoded by mitochondrial DNA. Pic2p and Hem25p help provide co-factors that are essential for complex IV assembly and function. Hem25p transports glycine that is used to make Heme and Pic2p mediates uptake of Cu^++^. TOM, translocase of the outer membrane; TIM23, translocase of the inner membrane 23; Oxa1p, mitochondrial inner membrane insertase.

In yeast and in humans, a link between the function of MCF members and COX biogenesis is emerging (Figure 5). For instance, a destabilizing pathogenic mutation in *SLC25A46* impairs OXPHOS and ascorbate/TMPD-dependent respiration and reduces steady state levels of complex IV subunits (Janer et al., 2016). These findings are consistent with a complex IV-specific assembly defect in *SLC25A46* mutant fibroblasts. At present, the mechanistic basis for the reduced steady state levels of complex IV subunits has not been determined. A role in the assembly of the OXPHOS machinery is perhaps not unexpected given that many SLC members provide substrates that serve as building blocks needed for processes such as mitochondrial DNA replication, transcription, translation, and/or post-translational assembly of protein complexes. However, it is surprising that these defects seem to specifically impact complex IV without significantly affecting the other OXPHOS complexes that are also built from subunits expressed from both the nuclear and mitochondrial genomes.

Recently, a role for the yeast ortholog of human SLC25A38, Hem25p, important for heme synthesis as a mitochondrial glycine importer, in the stability of respiratory complex proteins was tested (Dufay et al., 2017). While deletion of Hem25p compromises the steady state level of subunits of each respiratory complex, its absence is most detrimental to complex IV (Dufay et al., 2017). Intriguingly, the combined absence of Hem25p and Flx1p, the mitochondrial flavin adenine dinucleotide transporter (ortholog of human *SLC25A32*), further reduces the steady state level of subunits of the *hem25*Δ-affected respiratory complexes except complex IV (Dufay et al., 2017). These results are consistent with a model that Hem25p and Flx1p provide heme and FAD, respectively, which are required for the assembly of the respiratory chain complexes.

Knockdown of the two isoforms of *SLC25A3*, *SLC25A3-A*, and *SLC25A3-B*, in many different cell types results in reduced COX holoenzyme levels and activity (Boulet et al., 2017). When SLC25A3 is limiting, the steady state levels of COX4, a nuclear encoded subunit of COX, and mitochondrial copper are reduced (Boulet et al., 2017). Interestingly, COX4 abundance is rescued by copper supplementation (Boulet et al., 2017). Since copper is critical for the assembly of COX (Diaz, 2010; Baile and Claypool, 2013), defects in copper import are likely to impair COX biogenesis. Nevertheless, SLC25A3 function may be modulated and/or linked to the assembly of COX via a pathway that is presently unidentified (Boulet et al., 2017).

The absence of Aac2p in yeast leads to a specific reduction of COX activity while the activity of complex III is unaffected (Dienhart and Stuart, 2008). The reduced complex IV activity likely stems from lower steady state levels of its subunits in the absence of Aac2p. These findings are consistent with the impaired OXPHOS activity that occurs when Aac2p expression is decreased or ablated (Heidkämper et al., 1996; Muller et al., 1996; Müller et al., 1997; Claypool et al., 2008). Intuitively, the mechanistic basis for the reduced steady state levels of complex IV subunits could derive from a defect in any step in its biogenesis that is regulated/modulated by and/or dependent on the nucleotide transport function of Aac2p. Alternatively, the conserved AAC/ANT-RSC interaction may itself be critical for robust COX expression, assembly, and/or function Since the Aac2p and ANT1/ANT2 interactomes all included other SLC25 family members (Claypool et al., 2008; Lu et al., 2017), it is possible that these MCF–MCF interactions are critical for maintaining the abundance of metabolites that are needed for optimal mitochondrial translation and assembly of respiratory complexes.

Our group recently showed that in yeast regulation of complex IV activity by the Aac2p relies solely on the activity of the protein in a way that is mechanistically dependent on mitochondrial translation (Ogunbona et al., 2018). Indeed, similar to the complete absence of Aac2p (Dienhart and Stuart, 2008), an interaction-competent transport-dead mutant of Aac2p exhibits reduced complex IV activity, reduced levels of the complex IV holoenzyme, and reduced steady states levels of complex IV subunits, especially those encoded by mtDNA (Ogunbona et al., 2018). Interestingly, translation of complex IV subunits encoded by mtDNA is specifically decreased in the absence of Aac2p activity, either genetically imposed or due to the presence of AAC-specific inhibitors. In addition, when Aac2p is expressed but non-functional, the turnover of newly synthesized Cox3p is increased (Ogunbona et al., 2018). Together, these results establish a novel link between nucleotide transport and mitochondrial translation of complex IV subunits. A big question moving forward is whether this neoteric mechanism is evolutionarily conserved in mammals. Ongoing and future efforts focused on dissecting the functional significance of distinct aspects of the AAC/ANT interactome are likely to shed further insight into how it supports complex IV biogenesis.

## Perspectives

The MCF is an extensive cluster of transport proteins with essential biochemical and physiological functions. Individually and collectively, they have a broad role in intermediary metabolism and are pathophysiologically significant. In spite of this, there remain numerous fundamental gaps in our understanding of their cellular functions and if and how their activities are regulated. At the molecular level, the mechanistic basis for their role(s) in the regulation of many biologic processes, including OXPHOS, is still missing. Even for the best characterized MCF protein, Aac2p, new biology was recently uncovered suggesting that there is likely a ton of new biology to uncover in the biggest subfamily of SLCs. Future research should primarily focus on establishing the biological functions of each and every protein in the family. For proteins with assumed roles in mitochondrial energy production, it will be important to carefully dissect their molecular contributions to OXPHOS. Using the recently uncovered translational regulation of complex IV activity by Aac2p as a paradigm, upcoming research work should remain open-minded to the identification of unanticipated functional relationships that may exist between MCF members and other biological processes. Discovery of new members and novel functions of existing members will give us a better understanding of their pathophysiological roles. Long term, such information may guide development of effective therapeutic strategies to correct or better manage diseases resulting from their dysfunction. With recent advances in biomedical research such as the advent of gene editing technologies (e.g., CRISPR/Cas9), substantial and rapid progress in the field of MCFs is anticipated.

## Author Contributions

OO and SC reviewed the literature. OO wrote the initial draft of the review which also served as the Introduction of his Ph.D. thesis (Ogunbona, 2018), submitted to Johns Hopkins University School of Medicine, and generated all of the figures. SC provided guidance and edited the manuscript.

## Conflict of Interest Statement

The authors declare that the research was conducted in the absence of any commercial or financial relationships that could be construed as a potential conflict of interest.

## Abbreviations

AAC: ATP/ATP carrier
ANT: adenine nucleotide translocase
CiC: citrate carrier
COX: cytochrome *c* oxidase
IMM: inner mitochondrial membrane
MCF: mitochondrial carrier family
OMIM: Online Mendelian Inheritance in Man
OXPHOS: oxidative phosphorylation
ROS: reactive oxygen species
RSC: respiratory supercomplex
SLC: solute carrier
UCP: uncoupling protein
